# Acute effects of blood flow restricted aerobic exercise in type 2 diabetes mellitus

**DOI:** 10.1097/MD.0000000000039031

**Published:** 2024-08-02

**Authors:** Elif Şahin, Teslime Ayaz, Melda Saglam

**Affiliations:** a Güneysu Physical Therapy and Rehabilitation College, Recep Tayyip Erdogan University, Rize, Turkey; b Department of Internal Medicine, Faculty of Medicine, Bakircay University, Izmir, Turkey; c Faculty Of Physical Therapy and Rehabilitation, Hacettepe University, Ankara, Turkey.

**Keywords:** acute exercise, cycle exercise, endurance training, Kaatsu training

## Abstract

**Background:**

This study aimed to compare the acute effects of aerobic exercise performed with blood flow restriction (BFR), a novel method to increase exercise gains, with blood free flow (BFF) conditions in type 2 diabetes mellitus (T2DM).

**Methods:**

Fifteen individuals with T2DM performed BFF and BFR (40% of arterial occlusion pressure) cycling exercises 48 hours apart, at equal intensity (45% heart rate reserve) and duration (38 minutes). Systolic blood pressure (SBP), diastolic blood pressure (DBP), mean arterial pressure (MAP), blood glucose, heart rate, and muscle oxygen saturation (SmO_2_) were assessed before-after and during exercise sessions.

**Results:**

SBP, DBP, and MAP in the overload phase were higher in the BFR group than in the BFF group (*P* = .009, 0.031, and 0.013, respectively). Changes in blood pressure (∆SBP and ∆DBP) were similar between the BFF and BFR groups (*P* > .05), whereas ∆MAP differed (*P* = .016). Changes in blood glucose levels and heart rates were not significantly different between the groups. Although SmO_2_baseline was lower in the BFR group (*P* = .049), SmO_2_min and SmO_2_max did not differ significantly between the BFF and BFR groups.

**Conclusion:**

The similar decrease in blood glucose levels between the groups suggests that BFR exercise is favorable in terms of hypoglycemia. The higher blood pressure observed during the BFR exercise remained within safe limits. These results suggest that people with T2DM can safely perform BFR aerobic exercises; however, further studies are required.

## 1. 1. Introduction

Blood flow restricted (BFR) exercises involve wrapping a narrow cuff around the appendicular limb during exercise to restrict blood flow in a way that occludes venous flow, but only partially restricts arterial blood flow to the extremities, causing arterial turbulence. BFR was first developed in the late 1960s in Japan and was used for the next 40 years during aerobic and resistance exercises to enhance anaerobic and neuromuscular adaptations.^[1,2]^ After 2010, researchers’ interest in BFR training increased because the technique allowed muscle development even at very low loads without causing excessive stress on the muscular system, suggesting that metabolic stress is actually more important than mechanical stress for muscle growth.^[2]^ When applied in combination with aerobic exercise, even walking, BFR can increase strength and muscle hypertrophy in young people, which is normally not expected with such low-intensity aerobic training.^[3]^ This led to the application of BFR in many diseases where high-intensity exercise is not appropriate (e.g., cardiac diseases).^[4]^ Low-intensity BFR cycling (~40% peak aerobic power) leads to a lower cardiac response than high-intensity exercise (~85% peak aerobic power) and is better tolerated by a wider population.^[5,6]^ However, compared to the same intensity exercise performed without BFR, higher cardiac responses (blood pressure [BP] and heart rate) and rate of perceived exertion (RPE) have been observed in healthy subjects.^[7–10]^

Preservation of muscular structure and function is essential for metabolic health, especially in individuals with type 2 diabetes mellitus (T2DM), as muscle tissue is the most important site of glucose disposal.^[11]^ Thus, in addition to aerobic exercise, American Diabetes Association and American College of Sports Medicine recommend moderate to vigorous resistance exercise at least 2 to 3 days a week for individuals with T2DM as a way to increase muscle mass.^[12]^ However, it is also known that individuals with type 2 diabetes have many structural defects in their muscles (such as intramuscular adipose tissue deposition) and are more susceptible to injuries that can be caused by mechanical stress.^[13,14]^ Therefore, BFR exercise may be a promising method for individuals with T2DM to increase muscle strength and hypertrophy with less mechanical stress. However, most previous studies on the acute effects of this method have been conducted in healthy individuals, with the exception of a few that investigated acute responses to BFR combined with aerobic exercise in patients with chronic diseases, such as obesity and prehypertension.^[15,16]^ Thus, there is still a need for studies on the acute cardiovascular effects of BFR combined with aerobic exercise in populations with chronic diseases, especially vascularly compromised individuals. Individuals with T2DM are known to have impaired cardiovascular responses (e.g., exaggerated BP response) even during steady-state exercise.^[17,18]^ Therefore, this study aimed to compare the acute effects of BFR and blood free flow (BFF) cycling exercise performed at the same intensity (45% of heart rate reserve [HRR]) on BP, heart rate, muscle oxygenation, RPE, and blood glucose levels in individuals with T2DM.

## 2. Methods

This study was approved by the Clinical Research Ethics Board of Recep Tayyip Erdogan University (approval number: 26). All the participants provided written informed consent.

Fifteen individuals (n = 15) with T2DM were included in this study. The participants were recruited from the Recep Tayyip Erdogan University Research Hospital, Department of Internal Medicine. The individuals visited our laboratory on 3 nonconsecutive days, usually scheduled in the late morning and early afternoon (10:00–13:00). For each participant, measurements were performed at the same time of day at each visit, allowing 48 hours of rest between the measurement sessions. In addition, care was taken to measure the baseline (before exercise) blood glucose level exactly 3 hours after eating a normal caloric meal.

On the first day, each participant’s demographic information and clinical history (history of diabetes, medications used, and presence of other diseases) were obtained. The demographic characteristics of the participants are presented in Table 1. Body composition (body weight and body mass index) was measured using a bioelectrical impedance analysis system (Tanita DC-360, Tokyo, Japan), and an incremental exercise test was performed to determine the exercise capacity. The exercise test was performed using a cycle ergometer (Ergoselect 200, Ergoline GmbH, Germany) with 12-lead electrocardiography (QRS-Card Stress PC Digital ECG System; Pulse Biomedical Inc.). BP, heart rate, and muscle oxygenation were measured at the start, during maximal exercise, and at the end of the test. We used a ramp exercise test protocol with increments of 5 watts every 30 seconds, including 2-minute 25-watt warm-up and recovery periods.^[19]^ According to the results of this test, the intensity of the exercise sessions done in the second and third visits (both BFR and BFF conditions) was determined by finding the load corresponding to 45% of the patient’s HRR (~50-55% of maximal power-watts).

**Table 1:** Demographical, anthropometric, and disease characteristics of the patients.

Patient characteristics	n	%	Median (IQR)
Gender			
Female	6	40	
Male	9	60	
Age, yr			57 (49–59)
Body weight, kg			89.3 (82.3–105.89)
BMI, kg/cm^2^			32 (26.6–35.3)
Disease duration, yr			2 (0–14)
Medications			
β-blocker	2	13.3	
Insulin secretagogues	7	46.7	
Insulin therapy	5	33.3	

BMI = body mass index, IQR = interquartile range (Q1–Q3).

**Figure 1.**
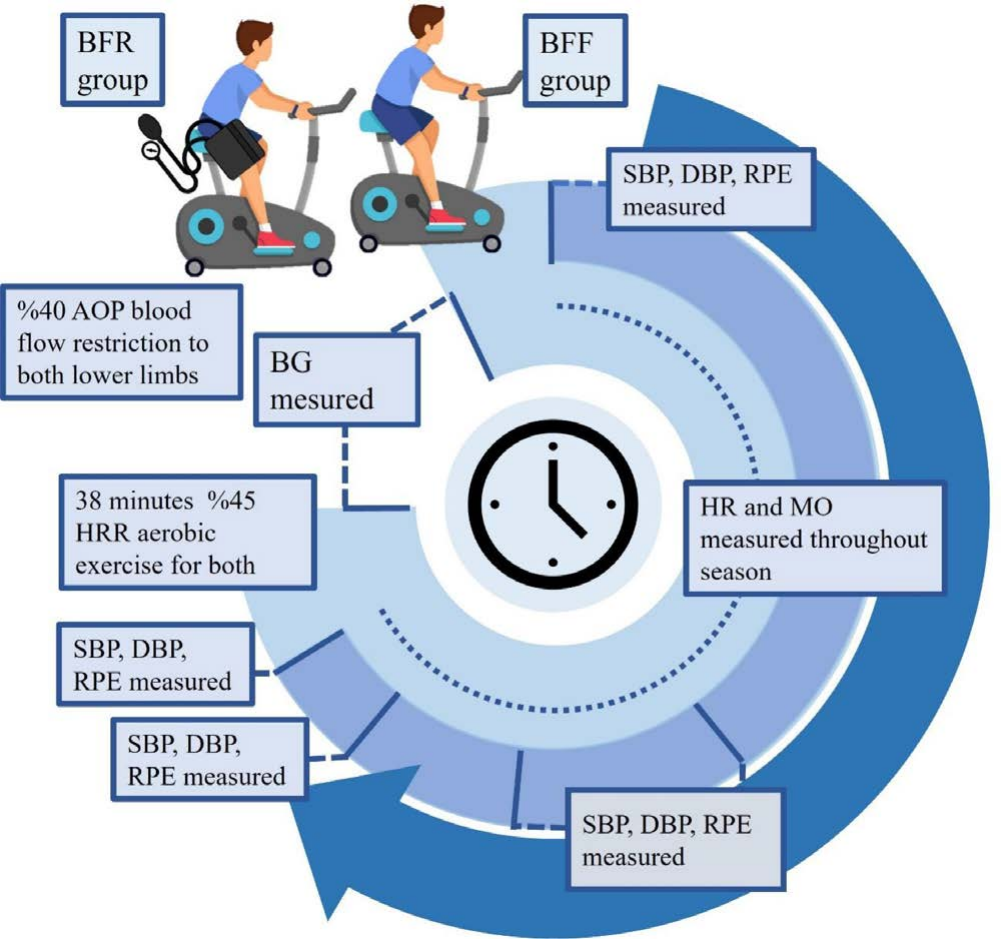
Study design and timeline of the exercise season of both groups. AOP = arterial occlusion pressure, BFF = blood free flow, BFR = blood flow restricted, BG = blood glucose, DBP = diastolic blood pressure, HR = heart rate, HRR = heart rate reserve, MO = muscle oxygenation, RPE = rate pressure product, SBP = systolic blood pressure.

At the second visit, all participants performed a BFF exercise session (continuous, steady-state) lasting 38 minutes, including warm-up and recovery. At the third visit, aerobic exercise with BFR was performed at the same intensity (45% of HRR) and duration (38 minutes total) as the BFF exercise session. Systolic BP (SBP), diastolic BP (DBP), blood glucose level, and RPE were assessed before and periodically during the overload phase, whereas muscle oxygenation and heart rate were monitored continuously throughout the exercise sessions. Heart rate, SBP, and DBP values were recorded by asking the patient to remain motionless for 1 minute before starting exertion (baseline), during the last 30 seconds of the overload phase, and within the 5-minute period after completion of the exercise session. RPE was evaluated using the modified Borg scale for dyspnea and leg fatigue (Fig. 1).

### 2.1. Muscle oxygenation

Muscle oxygen saturation (SmO_2_) data were measured over the gastrocnemius muscle of the dominant leg using a near-infrared spectroscopy device (Moxy; Fortiori Design LLC). The device was set to record values every 2 seconds. SmO_2_ data were compared between the BFF and BFR conditions minute-by-minute from preexercise rest to the end of the session. The total hemoglobin values at baseline and at the end of the test were also recorded and compared.

To observe changes in SmO2, measurements were recorded throughout the exercise session, from the preexercise rest period to the end of the postexercise recovery period. The baseline (SmO2base), lowest (SmO2min), and highest (SmO2max) values were determined, and relative (∆) values were obtained by calculating the difference between these values, expressed as SmO2base2−SmO2min, SmO2max−SmO2base, and SmO2max−SmO2min.

### 2.2. BFR method

**Figure 2.**
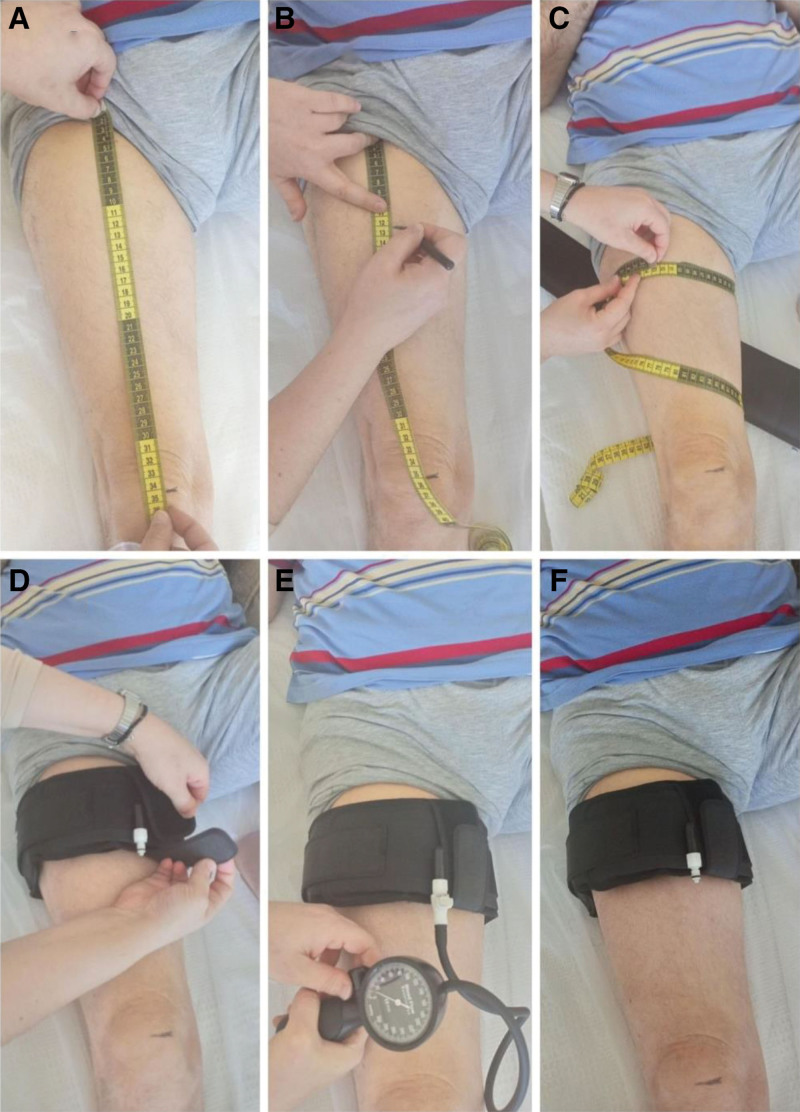
Determining the appropriate cuff pressure according to leg circumference. (A and B) Determination of 1/3 of the distance between the patella and inguinal ligament. (C) Measuring leg circumference. (D) Applying cuff. (E) Inflating the cuff to the appropriate pressure. (F) Automatic locking of the cuff stopper by removing the pump tip.

There are different methods for determining the pressure during BFR, including using a constant pressure (130–180 mm Hg) for all individuals, calculating the pressure individually by multiplying SBP by 1.3, or, more precisely, calculating the adequate pressure according to the arterial occlusion pressure (AOP).^[20,21]^ AOP is the pressure at which arterial flow is completely cut off, usually corresponding to 300 mm Hg or higher. During BFR, 40% to 80% of the AOP can be used. AOP can be also calculated individually based on SBP-DBP and extremity circumference.^[22]^ However, Loenneke et al showed that thigh circumference plays a key role in determining appropriate AOP.^[23]^ Loenneke et al calculated pressures corresponding to 40%, 50%, and 60% of AOP separately according to different limb circumferences.^[23]^ We used the formula of Loenneke et al to find the pressure corresponding to 40% of AOP for all individuals.^[23]^ Leg circumference was measured at the proximal 1/3 of the distance between the patella and the inguinal ligament. Then, the appropriate amount of pressure, according to each individual’s leg circumference, was applied to both thighs with a 10-cm pneumatic cuff device (Large 30-Inch STRAIGHT BFR Cuff, H^+^ cuff; Fig. 2).

### 2.3. Statistical analysis

Statistical analyses were performed using the IBM SPSS Statistics software (version 24.0; IBM Corp, Armonk). The heart rate, BP, muscle oxygenation measurements (SmO_2_ and total hemoglobin), blood glucose, and RPE values recorded during both exercise interventions were compared. The median was used as a measure of central tendency, and the interquartile range was used as a measure of dispersion. The Wilcoxon test was used for statistical analyses.

## 3. Results

Heart rate and BP values before exercise and during the overload phase and their ∆values (expressing the difference between initial and overload values) were compared between the BFF and BFR conditions. There was no statistically significant difference in the ∆heart rate values (*P* > .05), whereas SBP and DBP during the overload phase were significantly higher in the BFR exercise group than in the BFF exercise group (*P* < .05; Table 2).

**Table 2:** Cardiovascular, glycemic, and perceptional responses to BFF and BFR exercises.

	BFF, Median (IQR)	BFR, median (IQR)	*P*
Heart rate, bpm			
Baseline	82 (73–83)	81 (73–83)	.246
Exercise phase	116 (107–127)	116 (110–134)	.659
∆HR	35.5 (29–44)	35 (33–48)	.166
Blood pressure, mm Hg			
Baseline SBP	120 (110–135)	120 (110–130)	.602
Exercise phase SBP	150 (130–160)	160 (150–180)	.009*
∆SBP	20 (20–40)	50 (30–60)	.06
Baseline DBP	70 (70–80)	80 (70–80)	.943
Exercise phase DBP	80 (70–80)	80 (80–90)	.031*
∆DBP	0 (0–10)	10 (0–10)	.061
Baseline MAP	93.3 (83.3–100)	90 (83.3–96.6)	.959
Exercise phase MAP	103.3 (90–106.6)	113.3 (100–113.3)	.013*
∆MAP	8.3 (6.6–13.3)	20.0 (13.3–28.3)	.016*
Blood glucose, mg/dL			
Baseline	142 (127–184)	140 (112–210)	.842
Post	100 (91–119)	102 (92–128)	.460
∆BG	43 (33–72)	23 (14–70)	.589
RPE (modified Borg)			
Dyspnea	2.5 (1.25–3)	1.5 (0.5–2.75)	.236
Leg fatigue	4 (3.25–4)	4 (3.25–5.75)	.453

∆BG = final−baseline BG, ∆DBP = exercise phase−baseline DBP, ∆SBP = exercise phase−baseline SBP, BFF = blood flow free, BFR = blood flow restriction, BG = blood glucose, BP = blood pressure, DBP = diastolic blood pressure, HR = heart rate, MAP = mean arterial pressure, RPE = rate of perceived exertion, SBP = systolic blood pressure.

* *P* < .05.

### 3.1. Muscle oxygenation

**Figure 3.**
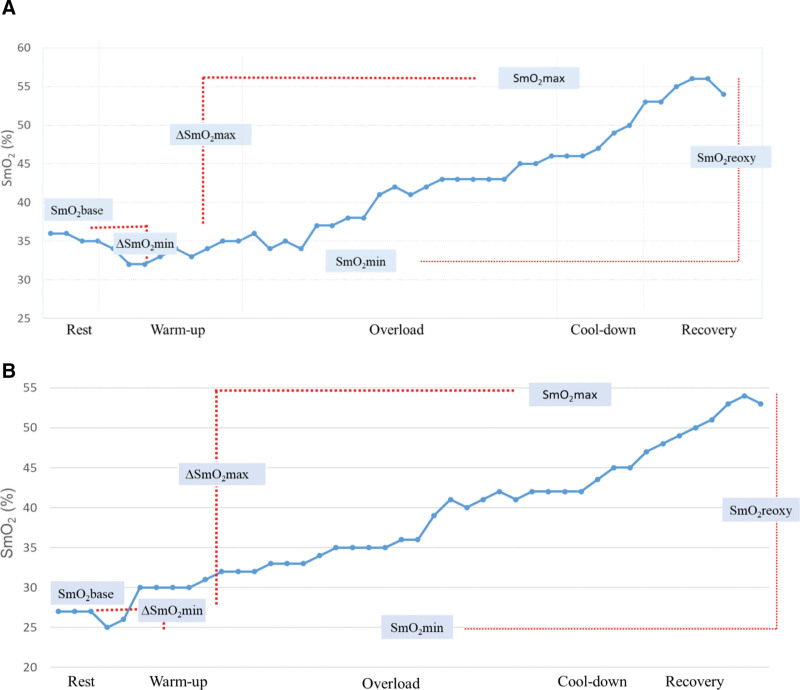
Muscle oxygenation (SmO_2_) median values and the parameters analyzed during the blood flow restricted (A) and blood flow free (B) exercise sessions. ∆SmO_2_max = SmO_2_max−SmO_2_base, ∆SmO_2_min = SmO_2_base−SmO_2_min, SmO_2_base = baseline muscle oxygen saturation, SmO_2_max = highest muscle oxygen saturation, SmO_2_min = lowest muscle oxygen saturation, SmO_2_reoxy = SmO_2_max−SmO_2_min.

The SmO_2_ parameters used are shown in the graph showing synchronous real-time median values under BFF and BFR conditions (Fig. 3). SmO_2_ tended to increase in both groups during the exercise period. The SmO_2_base value was significantly higher in the BFF condition than in the BFR condition (*P* = .049; Table 3).

**Table 3:** Comparison of muscle oxygenation measurements during the BFF and BFR exercise sessions.

	BFF exercise, median (IQR)	BFR exercise, median (IQR)	*P*
SmO_2_base, %	36 (27.25 to 49)	26.5 (19.75 to 49.75)	0.049*
SmO_2_min, %	32 (28.5 to 44.5)	25 (22 to 46)	0.515
∆SmO_2_min, %	3 (−0.5 to 14)	5 (2 to 10.5)	0.859
SmO_2_max, %	56 (42 to 61)	54 (38 to 69.75)	0.944
∆SmO_2_max, %	12 (7.5 to 21.5)	20 (14.75 to 28.5)	0.407
SmO_2_reoxy, %	19 (12 to 28)	27 (18.75 to 35)	0.173
tHb baseline, g/dL	12.77 (12.27 to 12.89)	12.77 (12.58 to 12.91)	0.283
tHb final, g/dL	12.43 (12.10 to 12.64)	12.51 (12.26 to 12.79)	0.386

* *P* < 0.05.

IQR = interquartile range, SmO_2_ = muscle oxygenation, tHb = total hemoglobin.

### 3.2. Adverse events

No serious adverse events indicating an ischemic event (e.g., ST-segment changes) were observed during the BFR exercise. Two individuals reported leg pain immediately under the BFR cuff; however, the pain resolved at the end of the session. Two individuals reported itching after cuff removal. All individuals reported widespread burning pain distal to the cuff, but this was expected because of the increased lactate accumulation compared with the BFF exercise condition. According to the American College of Sports Medicine, BP should not exceed 220/105 during endurance exercises for safety although a specific cutoff value is not defined for T2DM.^[24]^ In this study, the BP in the overload phase was below this value in all individuals performing BFR exercise.

## 4. Discussion

When we compared BFF and BFR exercises at the same intensity in the same individuals, there was no difference in heart rate values between the BFR and BFF conditions. The results were similar for ∆SBP and ∆DBP (relative values obtained by calculating the difference from the baseline) and blood glucose values. However, both SBP and DBP were higher when cycling with BFR although they were still within safe limits.

One of the most important findings of this study was the acute effect of BFR exercise on BP in diabetes, which is known to increase the risk of hypertension due to vascular complications. It is known that individuals, even those with normal BP at rest, may show an exaggerated BP response during exercise.^[25]^ Two previous studies demonstrated both higher SBP and DBP with low-intensity BFR cycling performed at an intensity corresponding to 40% of maximal oxygen consumption or 40% of peak power in healthy subjects.^[6–10]^ One of these studies compared BP during 2-minute bouts of cycling (40% of peak power) in BFR and BFF conditions and found that both SBP and DBP increased during all training bouts.^[6]^ In another study with longer exercise duration, Kumagai et al reported that SBP and DBP measured at minute 10 of a 30-minute cycling session were high although both decreased progressively after minute 10 until the end of the exercise session.^[10]^ Ozaki et al compared BP during 4-minute bouts of BFR cycling with BFF conditions at different exercise intensities (20%, 40%, and 60% of maximal oxygen uptake) and found SBP to be higher in BFR even at the lowest exercise intensity. However, DBP was found to be significantly higher in the BFR group only at the exercise intensity corresponding to 60% of maximal oxygen uptake.^[7]^

Few studies have been performed in patients with cardiovascular risk, and most have been performed with resistance exercises. Pinto et al investigated the acute effects of BFR with resistance exercise in patients with hypertension and showed that SBP increased from 146.2 ± 19.6 to 237.2 ± 33.2 mm Hg in BFR exercise versus an increase from 140.3 ± 18.2 to 192.7 ± 19.8 mm Hg in the non-BFR condition.^[26]^ The study most similar to ours was conducted by Karabulut and Garcia with people with obesity who performed 20-minute cycling exercises with 10-minute bouts. They found that BFR induced an approximate 12-mm Hg increase in SBP above non-BFR sessions.^[16]^ When we calculated the relative increase from baseline, the increase was 20 mm Hg in the BFF condition and 50 mm Hg in the BFR condition. However, the greater difference in our study is not surprising, as our patient group may have been more prone to hypertension than obese individuals.

In terms of heart rate, we observed similar increases in both groups. This result differed from those of previous studies, which demonstrated a higher heart rate with BFR cycling compared to the non-BFR condition.^[7,10,16]^ However, when we reviewed these studies, we found that baseline heart rate was also higher in the BFR group in 2 of the studies.^[7–10]^ The authors argued that the reduction in venous return would reduce stroke volume over time, which would increase heart rate.^[16]^ In another review, Queiros et al claimed that BFR has the capacity to exaggerate the metabolic stress provided by low-intensity aerobic training via hypoxia and occlusion of venous return and increasing the accumulation of metabolites.^[27]^ However, the pressure applied in all studies above was greater than ours. Therefore, the relatively lower occlusion pressure applied in our study may have been insufficient for these effects and the stimulation of receptors, resulting in a lack of a significant increase in the heart rate.

A similar decrease in blood glucose levels was observed under both exercise conditions in the present study. It is generally agreed that blood glucose is reduced with moderate-intensity exercise, while it is highly affected by other parameters (time and content of the last meal eaten).^[28]^ However, there are inconsistent results regarding the effects of exercise intensity on blood glucose reduction, with some studies showing more and others showing less decline with high-intensity exercise.^[29,30]^ This inconsistency is also seen in comparisons of different exercise types, especially when comparing resistance exercise with aerobic exercise. Some researchers have stated that resistance exercise results in lower glucose concentrations immediately following and up to 30 minutes after exercise compared to high-intensity interval aerobic exercise.^[31]^ Yardley et al compared resistance exercise with low-intensity exercise and concluded that plasma glucose levels decreased rapidly during aerobic exercise, resulting in significantly lower levels than in those with no exercise. However, decreases during resistance exercise were more gradual and of a smaller magnitude, with differences being significant only after 45 minutes of resistance exercise compared with the no-exercise session.^[32,33]^ These results are somewhat parallel with ours, as our study involved an exercise with an effect similar to that of resistance exercise. However, we only measured blood glucose levels immediately before and after exercise, indicating that we cannot ascertain whether BFR exercise causes a delayed but exaggerated reduction in blood glucose, similar to resistance exercise. It should also be noted that there is evidence showing that tissue hypoxia enhances the translocation of glucose transporter 4 to the cell membrane to facilitate glucose uptake into myofibers.^[34]^

Examining the SmO_2_ data, we initially noted a small drop in SmO_2_ followed by a progressive increase. The significant difference in baseline values between the 2 groups persisted during the warm-up period but then disappeared. SmO_2_ reflects the balance between oxygen supplied to and consumed by the muscle. The decrease in blood flow during the BFR condition at the beginning of exercise may have caused the SmO_2_ to be lower at baseline and during the warm-up period. However, as the load increases, the effect of oxygen supply on SmO_2_ may decrease and the effect of consumption on SmO_2_ may increase, meaning that the SmO_2_ value begins to reflect consumption rather than oxygen supply. Our study is similar to that of Niemeijer et al, which was conducted in patients with heart failure. In that study, patients performed constant-load cycling for 6 minutes at 80% of the anaerobic threshold.^[35]^ They experienced a rapid decrease in SmO_2_ at the beginning of exercise, followed by a gradual rise throughout the exercise session and a rapid rise during recovery. Unsurprisingly, there was more deoxygenation in that study at the beginning of exercise (−10.1%±6.1%) than in our study because of the different near-infrared spectroscopic devices used and the different patient groups, which were more prone to oxygen delivery deficits. Another reason for this small difference may be the sudden-onset loading phase performed without warming up in that study as opposed to our protocol, which included a properly gradual warm-up phase to prevent oxygen deficit.

### 4.1. Limitations

However, when interpreting cardiovascular responses, disease duration should also be considered. The median disease duration in our patients was 2 years, which can be regarded as a very short period in terms of vascular complications. Therefore, studies are needed in patients with longer disease duration and higher risk of microvascular and macrovascular complications to reach a definitive conclusion, considering that complications such as autonomic neuropathy may even change the heart rate during exercise. From this point of view, the fact that almost half (46.6%) of the individuals included in this study had a disease history of less than 1 year can be considered a major limitation.

Another limitation of this study is the subjective evaluation of muscle fatigue using only the modified Borg scale. Muscle fatigue can be assessed more objectively using electromyography or lactate measurements; however, these are invasive methods. Another factor that can affect fatigue is the applied BFR pressure. Mouser et al showed that relative pressures in the range of 40% to 60% of the AOP appear to elicit similar blood flow characteristics.^[36]^ However, since this study was conducted in healthy individuals, it is not known whether there will be a difference at pressures >40% of the AOP in individuals with vascular complications. Therefore, it is difficult to make a general estimation of these responses at a 40% AOP pressure, which can be considered low.

## 5. Conclusion

In this study of individuals with T2DM, BFR aerobic exercise was associated with higher SBP and DBP, higher mean arterial pressure in the loading phase, and a larger relative increase in mean arterial pressure from baseline than BFF exercise. However, considering that BP remained within safe limits during exercise and BFR has many positive effects, these increases can be ignored. When looking at the SmO_2_ value, it was seen that oxygenation falls at the beginning of the BFR exercise, but the difference disappears after the muscle pump is activated. In addition, the similar drop in blood glucose in both groups suggests that BFR may also be positive in terms of hypoglycemia, considering that the fear of hypoglycemia is a barrier to exercise in patients with diabetes. However, further studies with larger patient groups and different disease durations are needed to reach a definite conclusion.

## Author contributions

**Conceptualization:** Elif Şahin, Teslime Ayaz, Melda Saglam

**Data curation:** Elif Şahin, Melda Saglam

**Formal analysis:** Elif Şahin

**Funding acquisition:** Elif Şahin

**Investigation:** Elif Şahin, Teslime Ayaz

**Methodology:** Elif Şahin, Melda Saglam

**Project administration:** Elif Şahin

**Validation:** Elif Şahin

**Visualization:** Elif Şahin

**Writing—original draft:** Elif Şahin

**Resources:** Teslime Ayaz

**Supervision:** Teslime Ayaz

**Writing—review & editing:** Teslime Ayaz, Melda Saglam
